# Effects of breathing movement on the reduction of postural sway during postural-cognitive dual tasking

**DOI:** 10.1371/journal.pone.0197385

**Published:** 2018-05-29

**Authors:** Kohtaroh Hagio, Hiroki Obata, Kimitaka Nakazawa

**Affiliations:** 1 Sports Science Laboratory, Department of Life Sciences, Graduate School of Arts and Sciences, The University of Tokyo, Tokyo, Japan; 2 Department of Humanities and Social Sciences, Institute of Liberal Arts, Kyushu Institute of Technology, Fukuoka, Japan; Tokai University, JAPAN

## Abstract

An execution of cognitive processing interferes with postural sway during quiet standing. It reduces sway variability in young adults, but the mechanism is not clear. To elucidate the mechanisms, we focused on breathing in the present study. The purpose of this study was to clarify whether a decrease in postural sway amplitude during a postural–cognitive task is related to the change in breathing movement. The center of pressure (COP) was recorded via a force plate and the motion of leg joints (ankle, knee, and hip), and breathing movements were measured with a 3D motion capture system in quiet standing and standing with cognitive (mental arithmetic) task conditions. The change ratios of each variable from the quiet standing condition to the cognitive task were also calculated. It was shown that the MASt condition produced a significantly smaller RMS of COP displacement as compared to the QSt condition (p < 0.01). The results revealed that the breathing rate was faster and the amplitude of breathing movement smaller when subjects performed the cognitive task. A significant positive correlation (r = 0.75, p < 0.01) was found between the change ratio of breathing amplitude and the COP amplitude. The present results suggest that reduced standing postural sway during a cognitive task is related, at least in part, to a decrease in breathing amplitude.

## Introduction

In daily activities, humans control their standing or walking posture adequately in situations where cognitive processing is simultaneously required for thinking about something or talking with others. Previous studies reveal that postural control and cognitive processing influence each other, depending on the complexity of both tasks [1–3]. Some theories exist as to how cognitive processing affects postural performance [4]. One of the convincing models suggests that posture control and cognitive activity share a neural resource [3]. This model explains the degraded performance of one or both tasks when a postural–cognitive dual task is executed. Balance control impairment and body instability in older adults resulting from deficits in the allocation of attention have been observed during postural–cognitive dual tasking [1,3,4].

In young adults, it has been reported that cognitive activity reduces postural sway during quiet standing [5–10]. For example, it is known that performing suprapostural tasks, such as a visual search [8–10] or non-spatial working memory task [5–7], reduces sway amplitude. It has been suggested that in a visual search task, the stance can be modulated in ways that facilitate the performance of some suprapostural tasks [9]. To explain these results for a non-spatial working memory task, some researchers suggest that cognitive activity increases the capacity of neural resources through an increase in arousal level so that postural performance in dual task conditions could be improved [4]. Others have suggested that cognitive processing releases postural control from attentional focus and allows more automatic control processes [5,11]. However, the actual mechanisms for how cognitive activity reduces postural sway remain poorly understood.

In the present study, to approach the mechanisms of reduced postural sway during postural–cognitive dual tasks from another viewpoint, we focused on changes in breathing movements during cognitive activity. Breathing is known to affect postural sway and during standing [12–14] and to be affected by psychological stressor (e.g., cognitive task) through sympathetic nerve activation [15,16]. Greater amplitudes of COP displacement have been reported when subjects voluntarily increased their tidal volume [12], and smaller amplitudes of COP have been reported in an apnea condition [13], as compared to quiet breathing. Taken together, it is possible that postural sway and cognitive processing are related through breathing. If the variation in the breathing movement of the ribcage decreases, postural sway will decrease. Therefore, in the present study, we hypothesized that cognitive processing reduces postural sway due to changes in the breathing pattern. The purpose of this study was to clarify whether a decrease in postural sway amplitude during a postural–cognitive task is related to the change in breathing movement.

## Methods

### Subjects

Fifteen healthy young males participated in this study. The mean age, height, and weight of the subjects were 23.9 ± 3.0 years, 163.0 ± 6.4 cm, and 66.5 ± 8.7 kg, respectively. Subjects had no history of neurological, cardiopulmonary, or vestibular problems or pathologies of the lower limbs. The experimental procedures used in the study were in accordance with the declaration of Helsinki and were approved by the ethical standards of the committee on Human Experimentation at the Graduate School of Arts and Sciences, the University of Tokyo. All subjects gave their informed written consent after receiving a detailed explanation about the purpose, potential benefits, and risks involved in participating in the study.

### Procedure and measurement

Barefoot subjects were required to keep an upright stance on a force platform (Type 9281B; Kistler, Switzerland) with their eyes open and feet parallel at a 15 cm inter-heel distance. Subjects held their arms to the sides of their body while looking at a target placed 1.5 m in front of them at eye level. Two standing conditions, described in the following section, were observed. Subjects performed three 30 s trials under two standing conditions (described in the following section), with sufficient rest between trials, and these six trials were fully randomized. The ground reaction forces (GRFs) were recorded at a sampling rate of 1 kHz and stored on the computer for later offline analysis.

**Fig 1:**
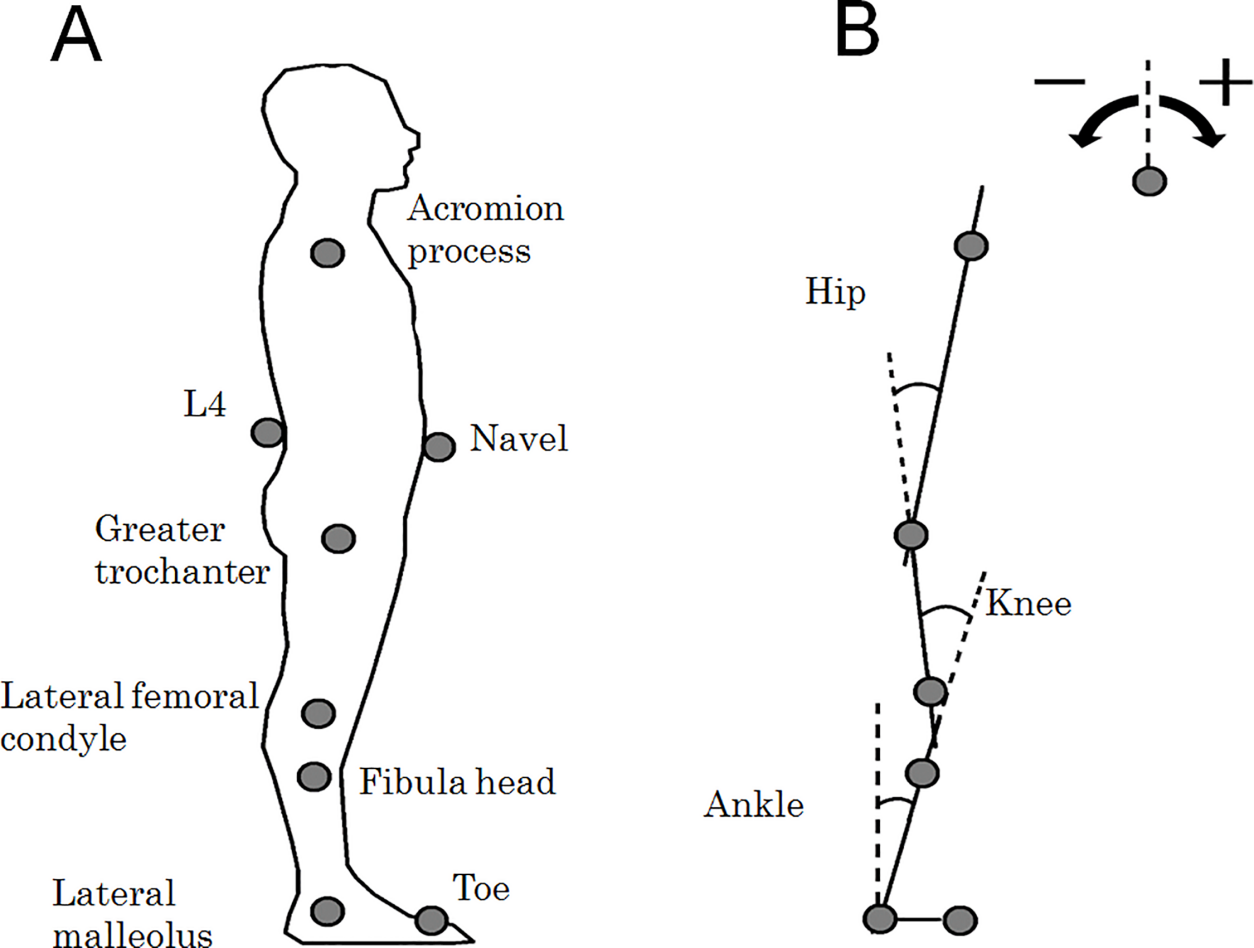
Experimental setup. (A) Marker placement: Eight reflective markers were placed over surface landmarks. The markers, except for L4 and the navel, were used to calculate the ankle, knee, and hip joint angles in the sagittal plane. The markers of L4 and the navel were used to calculate the abdominal breathing movement. (B) Definitions of joint angles: Joint angles were expressed as positive in the clockwise direction.

The kinematic data were also recorded in each trial at a sampling rate of 100 Hz to examine the interaction between whole-body fluctuation and breathing movement. The three-dimensional Cartesian coordinates of the markers were obtained with an optical motion capture system (OptiTrack V100R2; NaturalPoint, USA) composed of six infrared cameras in a semi-circular arrangement. Six reflective markers (5 mm diameter) were placed over surface landmarks (Fig 1A).

### Postural conditions

Two postural conditions were observed in this study. In the quiet standing (QSt) condition, subjects were asked to relax and maintain an upright stance. In the standing with mental arithmetic (MASt) condition, they were asked to count backward from randomly selected three-digit numbers in single-digit-number steps as fast and accurately as possible while standing quietly. At the end of the MASt trials subjects were asked to verbally report the final number. Before testing, subjects practiced the mental arithmetic task several times while sitting.

### Data processing and analysis

#### COP variables

From the measured GRFs, COP displacements in the anterior–posterior (AP) direction were calculated. The COP time series were filtered, with a cutoff frequency of 10 Hz, using a second-order low-pass Butterworth filter, and then the mean value of the trial was subtracted from it. Two COP variables, the root mean square (RMS) of COP displacement and the mean velocity (MV) of COP displacement, were calculated in the AP direction. The RMS of COP is representative of displacement-related measures, allowing us to estimate the overall standing postural performance, whereas the MV of COP is representative of velocity-related measures that provide information on postural corrections required to maintain postural stability [17,18].

The RMS and MV of COP were calculated using the following formulas:

$$RMS=\sqrt{\frac{1}{n}\sum_{i=1}^{n}{\left(x_{i}\right)}^{2}}$$

$$MV=\frac{1}{T}\sum_{i=1}^{n-1}\left|x_{i+1}-x_{i}\right|,$$

where *x_i_* denotes the COP displacement in the AP direction at a given instant, and T denotes the duration of the time series.

One subject, who did not show decreased COP variables, was excluded from analysis thereafter to show the relation between the decrease in postural sway amplitude during dual tasking and the change in thoracic movement caused by respiration.

#### Joint angles

The ankle, knee, and hip joint angles in the sagittal plane were calculated from the marker coordinates (Fig 1A) [19]. The definition of each joint angle is shown in Fig 1B. The joint angles are expressed as positive in the clockwise direction. Briefly, the ankle joint angle was calculated from the markers at the lateral malleolus and fibular head. The knee joint angle was calculated from the ankle joint angles and the markers at the lateral femora condyle and greater trochanter. The hip joint angle was calculated from the knee joint angle and the markers at the greater trochanter and acromion process. The joint angles were filtered, with a cutoff frequency of 5 Hz, using a second-order low-pass Butterworth filter. The standard deviations (SDs) of the joint angles were calculated to assess the amplitude of the angular displacement of each joint.

#### Breathing movements

Breathing movements were estimated based on the distance between the abdominal markers (L4 and the navel), because most of the subjects were using abdominal breathing. The displacement of breathing movement was low-pass filtered, with a cutoff frequency of 0.5 Hz, using a second-order low-pass Butterworth filter to clarify the trunk motion with breathing. The average breathing rate was calculated from the displacement of the abdominal breathing movement. Time points of maximal inspiration were estimated from the displacement, and the number of points was expressed in breaths per minute. The average breathing rate was calculated from the peak-to-peak times and expressed in breaths per minute. To estimate the amplitude of breathing movement, the SD of the displacement of breathing movement was calculated.

#### Change ratios of variables

To normalize calculated variables among the subjects, in each amplitude variable (COP, joint angle, and breathing), the change ratios were calculated for each subject. These ratios indicate the difference between the QSt and MASt conditions, that is, the rates of change from the baseline in each amplitude variable were obtained by the following equation:

$$change\;ratio=\frac{M_{MASt}-M_{QSt}}{M_{QSt}}$$

Accordingly, *M*_MASt_ and *M*_QSt_ are the mean values of each variable during the MASt (*M*_MASt_) and QSt (*M*_QSt_) conditions, respectively.

### Statistical analysis

The average value across three trials was used as a representative value of COP variables and breathing movements. All statistical analyses were performed by the MATLAB program (version 2015b, Mathworks; Natick, USA). Paired t-tests were performed to examine the effects of two task conditions (QSt and MASt) on each variable, since Shapiro–Wilk test suggested that the data were normally distributed. Pearson's correlations were used to explore relationships between the change rates of the postural sway amplitude (i.e., the RMS of COP displacement and the SD of each joint) and the breathing amplitude. The correlation coefficient (r) indicates the strength of the relationship (classification of its strength was set as weak: 0.00–0.40; moderate: 0.41–0.70; or strong: 0.71–1.00). The regression line is estimated from simple linear regression. The significance level was set at α = 0.05. The effect size (ES) is reported as Cohen’s d, corresponding to the contrasting QSt and MASt conditions.

## Results

### COP measures

The mean values of COP variables are shown in Table 1. A paired t-tests revealed that the MASt condition produced a significantly smaller RMS of COP displacement as compared to the QSt condition (p < 0.01). No significant difference in the MV was observed (p = 0.44).

**Table 1:** Mean values and standard errors of COP measurements in the quiet standing (QSt) and standing with mental arithmetic (MASt) conditions.

COP measurements	QSt		MASt		t	p	d
	Mean	SE	Mean	SE			
RMS AP (mm)	3.47	0.66	2.49	0.43	4.14	< 0.01	1.02
MV AP (mm/s)	6.1	0.86	5.87	1.05	0.79	0.44 (n.s.)	0.14

Note. RMS: root mean square; AP: anterior-posterior; d: effect size

### Breathing measures

The mean values of the respiratory variables are shown in Table 2. A paired t-test revealed that the respiratory rate in the MASt condition was faster than that in the QSt condition (p < 0.01). The amplitude of the respiration movement in the MASt condition was smaller than that in the QSt condition (p < 0.05).

**Table 2:** Mean values and standard errors of breathing measurements in the quiet standing (QSt) and standing with mental arithmetic (MASt) conditions.

Breathing measurements	QSt		MASt		t	p	d
	Mean	SE	Mean	SE			
Breathing rate (times/min)	16.04	1.77	19.76	1.93	-5.55	< 0.01	1.16
Abdominal movement SD (cm)	2.39	0.75	1.58	0.40	2.42	< 0.05	0.78

Note. SD: standard deviation; d: effect size

### Correlation between change rates

**Fig 2:**
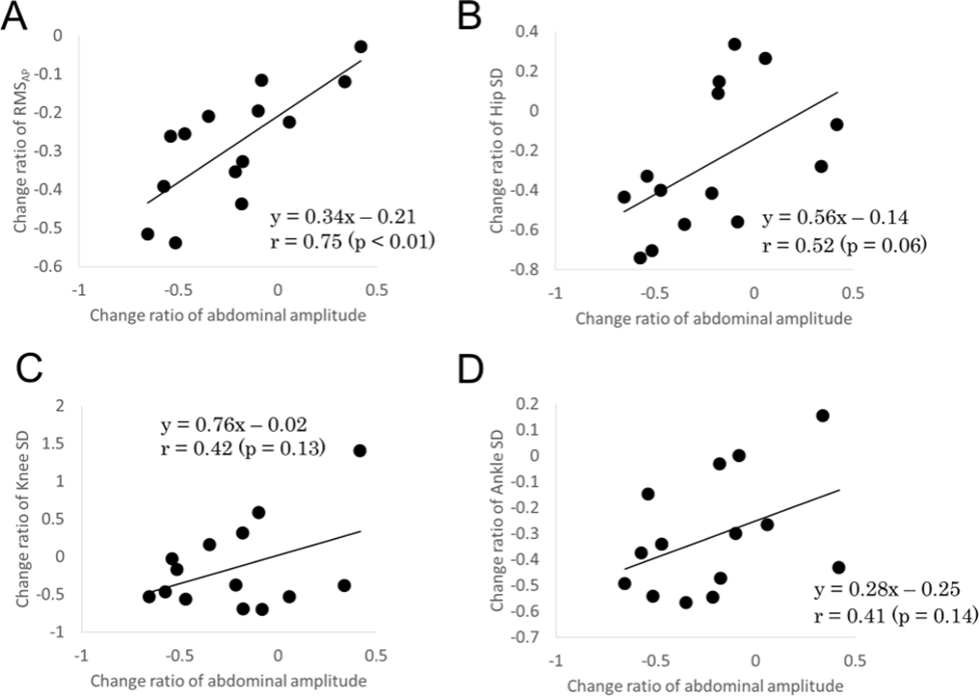
Relation between the ratio changes in breathing amplitude and the ratio changes in (A) the root mean square (RMS) of COP displacement in the anterior–posterior (AP) direction, (B) the standard deviation (SD) of hip movement, (C) the SD of knee movement, and (D) the SD of ankle movement.

The regression line, the correlation coefficient value, and its significance are presented in Fig 2. A significant positive correlation was found between the breathing amplitude change ratio and the COP amplitude change ratio (r = 0.75, p < 0.01). No significant correlation was found between the change rate of the respiratory amplitude and the SD of ankle or knee and hip joint movement change ratios (ankle, r = 0.41, p = 0.14; knee, r = 0.42, p = 0.13; hip, r = 0.52, p = 0.06).

## Discussion

The purpose of this study was to clarify whether a decrease in postural sway amplitude during a postural–cognitive task is related to the change in breathing movement. The results showed that standing postural sway decreased in almost all subjects (14 of 15 subjects) when they performed the mental arithmetic task as compared to simple quiet standing. This is consistent with previous studies [5–7]. At the same time, the breathing rate increased and the breathing amplitude decreased during the mental arithmetic task. In addition, there was a significant positive correlation between the change ratios of breathing and the COP amplitude. To our knowledge, this is the first study showing that reduced standing postural sway during cognitive tasks is related to changes in breathing amplitude.

The increase in breathing rate during a mental arithmetic task is consistent with results obtained while sitting in previous studies [15,16,20,21]. The enhancement of sympathetic nerve activity is the most probable explanation, since increased heart rates were reported along with increased breathing rates in a previous study [15]. On the other hand, no studies have reported a decrease in breathing amplitude during a mental arithmetic task while standing. It is possible that reduced breathing amplitude is specific to the execution of a mental arithmetic task during standing.

Some researchers have suggested the interdependence of breathing movement and postural sway during the experimental manipulation of breathing [12,14]. Hodges et al. [12] showed greater amplitudes of COP displacement and joint movements when subjects voluntarily increased their tidal volume, as compared to quiet breathing. On the other hand, Caron et al. [13] reported that apnea decreased areas (90% confidence ellipse) of the COP and the center of gravity as well as the COP velocity, as compared with quiet breathing. Bouisset and Duchene [22] reported that cross-correlation between breathing and COP increases significantly from 0.09 when standing to 0.16 when sitting. This is likely because there is a reduction in the number of segments in the kinetic chain that could counteract the perturbation from breathing. It has also been shown that lower limb joint movements are coherent with breathing (>0.5), and it has been suggested that breathing-induced trunk movement is mostly compensated by the movement of body segments, particularly the hip joint [12]. Therefore, a decrease in breathing-induced trunk movement as a disturbance might result in a decrease in hip joint fluctuation. Actually, our results showed that there tended to be a positive correlation between the change in abdominal variation and the change in hip joint fluctuation. It is suggested that a decreased postural disturbance through reduced breathing amplitude may reduce the amplitude of COP displacement.

## Limitation

There are some limitations to our investigation. The abdominal amplitude does not justify all breathing movement. Although the results of this study suggested that abdominal changes accompanying breathing were related to changes in postural sway amplitude, whether a change in the abdomen caused a change in the amplitude of postural sway needs further consideration. The methodology used in this study cannot directly verify the causal interaction of breathing with postural sway. Further investigation is required to clarify this issue. In this study, performing a mental arithmetic task reduced postural sway during quiet standing in almost all subjects. However, it may not necessarily coincide with improvements in postural stability. Several studies have shown a disadvantage in performing cognitive tasks when postural perturbation is applied experimentally [23,24]. For example, Little and Woollacott [24] reported that the performance of postural recovery from surface perturbation deteriorated when a cognitive task requiring working memory was added during standing. It is possible that the central nervous system (CNS) sacrifices flexibility of postural control in return for concentrating on cognitive tasks during quiet standing. Further research is necessary to reveal how the CNS prioritizes the execution of two tasks that interfere neurologically.

In this study, the distance between foots (i.e. base of support) were same (15 cm) for all subjects. A same size of the base of support can promote a different postural sway pattern between subjects with different heights. It may affect the present result. In addition, all subjects participating in this study were male due to measurement constraints. Gender differences is possible to affect the modulation of breathing patterns, since female subjects may have different breathing pattern. Future research needs to consider the individual anthropometric characteristics and gender difference.

## Conclusion

In conclusion, the present results suggest that a decrease in standing postural sway during a postural–cognitive dual task is related, at least in part, to a decrease in breathing amplitude in male subjects. The results imply mechanisms for cognitive processing to affect postural sway.
